# Vowel and consonant confusions from spectrally manipulated stimuli designed to simulate poor cochlear implant electrode-neuron interfacesa)

**DOI:** 10.1121/1.4971420

**Published:** 2016-12-20

**Authors:** Mishaela DiNino, Richard A. Wright, Matthew B. Winn, Julie Arenberg Bierer

**Affiliations:** 1 Department of Speech and Hearing Sciences, University of Washington, 1417 NE 42nd Street, Box 354875, Seattle, Washington 98105, USA; 2 Department of Linguistics, University of Washington, Guggenheim Hall, Box 352425, Seattle, Washington, 98195, USA

## Abstract

Suboptimal interfaces between cochlear implant (CI) electrodes and auditory neurons result in a loss or distortion of spectral information in specific frequency regions, which likely decreases CI users' speech identification performance. This study exploited speech acoustics to model regions of distorted CI frequency transmission to determine the perceptual consequences of suboptimal electrode-neuron interfaces. Normal hearing adults identified naturally spoken vowels and consonants after spectral information was manipulated through a noiseband vocoder: either (1) low-, middle-, or high-frequency regions of information were removed by zeroing the corresponding channel outputs, or (2) the same regions were distorted by splitting filter outputs to neighboring filters. These conditions simulated the detrimental effects of suboptimal CI electrode-neuron interfaces on spectral transmission. Vowel and consonant confusion patterns were analyzed with sequential information transmission, perceptual distance, and perceptual vowel space analyses. Results indicated that both types of spectral manipulation were equally destructive. Loss or distortion of frequency information produced similar effects on phoneme identification performance and confusion patterns. Consonant error patterns were consistently based on place of articulation. Vowel confusions showed that perceptions gravitated away from the degraded frequency region in a predictable manner, indicating that vowels can probe frequency-specific regions of spectral degradations.

## I. INTRODUCTION

Despite the success of cochlear implants (CIs) in restoring auditory perception, CI users exhibit a wide range of performance on speech perception tasks. Accurate speech recognition depends on adequate spectral resolution, or the ability to resolve frequency components in a speech signal. Differences among CI users' spectral resolving capabilities may thus contribute to the variability in CI user speech perception scores. Poor interfaces between CI electrodes and auditory neurons, which result from degeneration of adjacent neurons or suboptimal placement of the electrode array, could result in loss or distortion of the spectral information transmitted through those particular channels. Importantly, that spectral distortion may be localized to a specific range of frequencies. The present study implemented a vocoder simulation in normal hearing (NH) listeners to determine the influence of specific regions of frequency distortion on vowel and consonant recognition performance and confusions. Spectral degradations mimicked the negative effects of suboptimal electrode-neuron interfaces on transmission of particular frequencies within a CI. These spectral manipulations should decrease overall phoneme identification performance and alter confusion patterns. Vowels will be particularly affected because their identification relies heavily on the resolvability of distinct frequency regions. The goal of this study was to understand and predict the patterns of phoneme perception errors resulting from degradation of specific frequencies, which may contribute to decreased speech identification scores of CI users with suboptimal electrode-neuron interfaces.

The present study utilized a channel vocoder, which is a system of sound coding that transmits auditory signals via their simplified amplitude envelopes across frequency bands (Dudley, 1939); this is an essential part of modern cochlear implant processing (Loizou, 2006). In vocoder processing, an incoming signal is analyzed by a series of bandpass filters, from which the time-varying amplitude envelope is extracted through half-wave rectification and low pass filtering. The resulting signal is applied to a carrier with corresponding frequency bandwidth. Vocoders are widely used in experiments investigating spectral degradation of speech signals (e.g., Shannon *et al.*, 1995; Dorman *et al*., 1997) and many recent studies have used vocoder processing to model conditions of poor spectral resolution specifically resulting from spread of excitation in the cochlea (e.g., Litvak *et al.*, 2007; Bingabr *et al*., 2008; Winn *et al*., 2015; Won *et al.*, 2015). However, while most vocoder studies have applied spectral distortion broadly across the spectrum, CI users with poor electrode-neuron interfaces may have particular regions of frequency degradation. The present study thus degraded specific frequency regions to simulate the effects of region-specific, suboptimal electrode-neuron interfaces on vowel and consonant perception in NH listeners.

In previous vocoder studies and others that have manipulated speech stimuli, identification performance decreases dramatically when the frequency components of speech sounds, particularly of vowels, are distorted (ter Keurs *et al*., 1992). Spectral and spectrotemporal characteristics are critical to differentiating many classes of speech sounds (ter Keurs *et al*., 1992). Accordingly, some common measures of spectral resolution (e.g., the ability to discriminate spectral ripple signals with different phases) are related to speech recognition scores of NH and hearing impaired listeners (e.g., Henry *et al*., 2005; Litvak *et al.*, 2007). Frequency resolution is especially difficult for individuals with CIs. Compared to the continuous receptive area on the basilar membrane creating excitation among thousands of receptor inner hair cells in a healthy human cochlea, CIs contain only 12 to 22 electrodes with which to transmit frequency information, limiting the spectral resolution for listeners with these devices. Consequently, speech recognition in quiet and noise is generally poorer in CI users than in NH listeners (cf. Friesen *et al.*, 2001).

For CI users, spectral-resolving capabilities are limited not only because of the small number of frequency processing channels, but also because of the interaction of current between channels (White *et al*., 1984). However, some perform better than others on tests of recognition of words (Dorman *et al.*, 1989; Gifford *et al*., 2008) and sentences (Koch *et al.*, 2004; Firszt *et al.*, 2004) in both quiet and noise. This occurs even within groups of CI users with the same implant type and signal processing strategy. While many factors such as duration of deafness, age at implantation, and cognitive abilities have been associated with variability in CI users' speech recognition performance (see Holden *et al.*, 2013, for review), variation in quality of spectral transmission among the channels in an individual's implant is also a likely contributor to the observed across-listener variance in speech perception scores. Each electrode in the array transmits a specific frequency band from the input signal, mimicking the tonotopic organization of a healthy cochlea. Because of the importance of spectral cues for vowel and consonant distinctions, loss or distortion of information from even one channel can potentially decrease speech recognition scores. Neighboring channels whose spread of current stimulates overlapping neural populations also distorts the perceived frequency spectrum. The degree of channel interaction within the implant has been found to account for a large amount of variance in CI user speech identification performance (Stickney *et al.*, 2006).

Within a single implanted array, certain electrodes may stimulate the auditory nerve less effectively than others due to suboptimal interfaces between the electrodes and the auditory neurons, resulting in degraded transfer of spectral information. A poor interface can occur as a result of electrodes placed relatively more distant from auditory neurons (Finley *et al.*, 2008; Holden *et al.*, 2013) or degeneration or death of those neurons (Miura *et al.*, 2002). Spectral information transmitted through channels with such poor interfaces either does not reach the auditory nerve, or, if the current for that electrode is increased to elicit an auditory percept, is likely distorted due to channel interaction (for review, see Bierer, 2010).

To characterize CI channels with suboptimal interfaces, Bierer (2007) utilized focused electrical stimulation to target specific neural populations, and found that higher levels of focused electrical current were required to reach auditory perception thresholds in some channels of a user's implant than for other channels. Since voltage decreases with distance from the electrode (Jolly *et al*., 1996), high thresholds suggest some electrodes are distant from auditory neurons either because the electrodes are positioned near the lateral wall of the cochlea or the neurons near them had degenerated (Bierer, 2010; Long *et al.*, 2014). Subsequently, Bierer and Faulkner (2010) observed that channels with elevated focused thresholds had broad psychophysical tuning curves, indicating poor frequency selectivity and providing further evidence of the connection between elevated focused thresholds and poor electrode-neuron interfaces. However, even after identification of such channels, it is not clear how poorly situated electrodes lead to specific errors in speech perception. Since each CI electrode channel transmits a particular part of the frequency spectrum of a sound to the auditory nerve, suboptimal electrode-neuron interfaces should result in a loss or distortion of *predictable* frequency components. This study utilized a vocoder simulation of poor electrode-neuron interfaces with NH listeners to examine the perceptual consequences of such degraded frequency regions on vowel and consonant identification.

Of the numerous studies that have used vocoder processing, only a limited set have investigated situations that reflect specifically poor regions of stimulation. Shannon *et al*. (2002) and Kasturi *et al*. (2002) simulated regions of CI user spiral ganglion neuron loss in NH listeners by setting the output of particular bandpass filters to zero to produce spectral “holes” in speech signals. The size and region of the spectral holes were varied. In addition, Shannon *et al*. (2002) reallocated frequency information to other channels in some vocoder conditions, and also turned off or reallocated stimulation of specific electrodes in CI listeners' implants to correspond to NH listening conditions. In both studies, and for CI and NH listeners, recognition of speech sounds was found to decrease with increasing size of the spectral hole. Shannon *et al*. found that recognition of vowels, consonants, and sentences did not differ between conditions of dropping or reallocation, suggesting that reallocation of the dropped frequency information did not improve speech intelligibility and may have instead distorted the frequency spectrum due to frequency-to-place mismatch. Kasturi *et al*. (2002) also performed an analysis of the perceptual weight, or relative importance for phoneme identification, of each manipulated channel. They found equal perceptual weighting across all channels for consonants but varying weights across channels for vowels, indicating that certain frequency regions are more important for accurate vowel recognition than others. Similarly, Throckmorton and Collins (2002) created low-, mid-, and high-frequency spectral holes as three of several vocoder manipulations with NH listeners and observed that the low-frequency loss was more detrimental to vowel and consonant identification scores than were the mid-frequency and high-frequency losses, respectively. Further, the low-frequency manipulation decreased vowel identification performance much more than consonant identification. Results from these vocoder studies demonstrate that a loss or distortion of spectral information through electrode channels can greatly impact overall speech perception scores. Additionally, certain channels may affect recognition of vowel sounds to a greater extent than other channels. Importantly, it is not yet clear whether frequency-specific spectral distortions result simply in increased errors, or in *predictable* errors. This study will test the hypothesis that phoneme error patterns are predictable based on the region of distortion. If this hypothesis is supported, it might be possible to use these error patterns in conjunction with other measures to learn about the contributions of putatively poor electrode-neuron interfaces to vowel and consonant perception.

The current set of experiments expands on previous work by examining speech confusion patterns to determine how degradation or loss of spectral information through particular channels affects the perception of specific speech sounds. Vowels were chosen as a focus because well-characterized spectral peaks called formants contrast these basic units of speech. The acoustics of vowels can thus be easily exploited to better understand the auditory system. Vowels, then, are the most appropriate stimuli to determine how specific frequency distortions influence phoneme perception. This study will also test the hypotheses that vowel confusion patterns can corroborate the location of CI suboptimal electrode-neuron interfaces that are predicted by an individual's focused threshold patterns. This knowledge can lead to better understanding of why CI user speech errors occur, what kind of errors occur, and the perceptual consequences of spectral distortion.

This study refers to vowel contrasts in terms of vowel *quality*, so as to describe the spectral vowel contrasts of North American English (as opposed to vowel *duration* contrasts in languages such as Japanese; for example, Hirata and Tsukada, 2009). While duration can aid in English vowel identification (Ainsworth, 1972), its contribution is negligible when spectral cues are available (Hillenbrand *et al.*, 1995). Accurate vowel recognition depends strongly on one's ability to resolve formants, particularly the first, second, and third formants (abbreviated as *F*1, *F*2, and *F*3) and to perceive the relationships between these formants within a vowel. Formants are observed as steady-state resonant frequencies (e.g., Peterson and Barney, 1952; Hillenbrand *et al.*, 1995), and time-varying changes in formants within a vowel signal (Nearey and Assmann, 1986; Hillenbrand and Nearey, 1999; Assmann and Katz, 2005), both of which are relatively more difficult to perceive by listeners with CIs, leading to relatively greater use of durational cues by this population (Winn *et al*., 2012). While prior studies have found that degrading or shifting frequency information decreases vowel identification scores (e.g., Fu and Shannon, 1999), vowel perception confusion patterns resulting from frequency-specific spectral manipulation had yet to be examined.

This study also included consonant stimuli for comparison to vowels because consonant recognition is more robust to spectral distortion (ter Keurs *et al.*, 1992; Xu *et al*., 2005) on account of their accompanying distinctive cues in the temporal domain. Consonants contrast among place of articulation (where they are produced in the oral cavity), manner of articulation (how the airflow is constricted in the vocal tract), and voicing (ostensibly whether or not the vocal cords vibrate during production). Perception of the place feature depends primarily on spectral information, whereas manner and voicing contrasts can be identified primarily by temporal cues. Thus both spectral and temporal cues can be used for accurate consonant identification. Because of the importance of spectral cues for perception of place of articulation, prior studies have found that consonants were most likely to be confused with others of the same manner and voicing but different place of articulation under conditions of frequency distortion (Dorman *et al.*, 1997) and in noise (Miller and Nicely, 1955).

While the very basic tasks of vowel and consonant perception are not the same as tests of more global speech perception abilities, spectral manipulation of phonemes can be well controlled, which is of particular interest to this study. Vowel and consonants can be degraded in more specific ways than more complex speech sounds (e.g., entire words or sentences), allowing for a better understanding of speech processing and categorization.

The present study used confusion matrices to examine the patterns of vowel and consonant identification errors resulting from particular manipulations of the frequency spectrum. These matrices indicate the number of times each phoneme was correctly identified and which phoneme(s) it was confused with when incorrectly identified. CI users' patterns of vowel and consonant confusions indicate the distinguishing features of these speech sounds that are not adequately transmitted through the implant (Remus *et al*., 2007). For example, Sagi *et al*. (2010) utilized a model of vowel perception to predict individual CI user's vowel confusion patterns based on the quality of transmission of steady-state formant cues through the implant. Therefore, to use phoneme error patterns to identify suboptimal electrode channels, the relationship between frequency-channel allocation and phoneme acoustics must be explored. In this study, systematic examination of vowel and consonant error patterns resulting from loss or distortion of spectral information was conducted using three methods: sequential information transmission analysis (SINFA; Wang and Bilger, 1973), perceptual distance analysis (e.g., Shepard, 1972), and perceptual vowel space analysis. These analyses progressively provide a more detailed understanding of the effects of spectral distortion. Together, these methods exploit the well-established history of linguistic feature analysis, psychoacoustic exploration, and visualization. Comparison of CI users' phoneme confusions to those made by NH listeners in this experiment support the hypothesis that subject-specific phoneme perception errors can help to interpret the perceptual consequences of suboptimal CI electrode-neuron interfaces.

## II. METHODS

### A. Subjects

Twelve adult NH listeners (6 male) between the ages of 21 and 29 years (mean age = 25.2 years) were recruited from the University of Washington campus and surrounding community to participate in this study. Subjects were native speakers of American English and underwent a screening to verify hearing at 20 dB hearing level across frequencies from 250 to 8000 Hz. All subjects gave written informed consent and were compensated for their participation. Experimental procedures were approved by the University of Washington Human Subjects Division.

### B. Stimuli

Ten vowels in /hVd/ context (/i/, “heed”; /ɪ/, “hid”; /eɪ/, “hayed”; /ɛ/, “head”; /æ/, “had”; /ɑ/, “hod”; /u/, “who'd”; /ʊ/, “hood”; /oʊ/, “hoed”; /ʌ/, “hud”) were presented under various conditions. Vowel stimuli were recorded from one male and one female talker from the Pacific Northwest to match the region that the subjects were recruited from, as regional dialect has been found to influence recognition of vowel sounds (Wright and Souza, 2012). A head-mounted close talking microphone was used to record vowel sounds in a double-walled sound-treated booth. Recordings were digitized at 44 100 Hz using a 16 bit quantization rate and were resampled to 22 050 Hz. Original stimuli were filtered from 60 to 10 000 Hz using a Hanning filter with a slope of 100 Hz to eliminate proximity effects. This study also used sixteen consonants in /aCa/ context (/p/, “aPa”; /t/, “aTa”; /k/, “aKa”; /b/, “aBa”; /d/, “aDa”; /ɡ/, “aGa”; /f/, “aFa”; /θ/, “aTHa”; /s/, “aSa”; /ʃ/, “aSHa”; /v/, “aVa”; /z/, “aZa”; /dʒ/, “aJa”; /m/, “aMa”; /n/, “aNa”; /l/, “aLa”) naturally spoken by a male talker (stimulus materials were the same as those used by Shannon *et al.*, 1995, which were created by Tyler, Preece, and Lowder at the University of Iowa Department of Otolaryngology, 1989.).

### C. Vocoder processing

The processing of speech stimuli was designed to simulate that of CI Fidelity 120 processing with the same frequency band allocations as those used in most Advanced Bionics devices (Advanced Bionics Corp., Valencia, CA). Speech stimuli were digitally sampled at 17 400 Hz and divided into 15 contiguous pseudo-logarithmically spaced frequency bands from 250 to 8700 Hz. The square root of the total energy in each channel was calculated to compute the envelope and the resulting signal was low-pass filtered at 68 Hz. The envelope from each channel was used to modulate a noise band with a center frequency equal to that of the corresponding channel. Filter output slopes were set at 30 dB/octave. This filter slope was selected to bring the performance of NH listeners to the range of better-performing CI listeners (Litvak *et al.*, 2007). The modulated noise bands were summed and presented to subjects through speakers in a sound-attenuating booth.

**FIG. 1.**
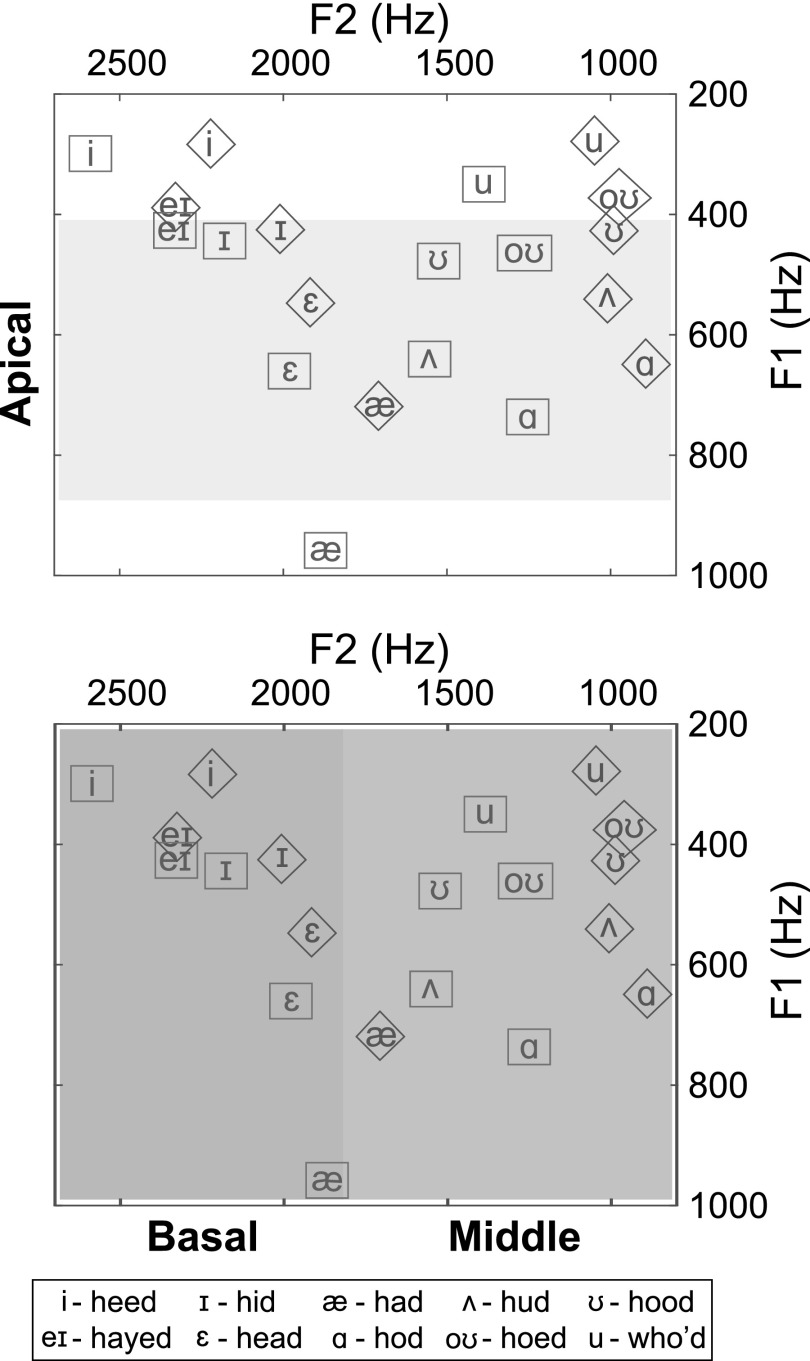
(Color online) Vowel space of stimuli. First formant frequencies are shown on the ordinate and second formant frequencies are on the abscissa. Formants shown were measured at the midpoint of each vowel. Shaded areas indicate manipulated frequency regions. Male talker vowels are enclosed in diamonds and female talker vowels are in rectangles. Plots show that the formant frequencies for each vowel are different between the male and female speaker and vocoder conditions will therefore affect male and female vowels in different ways. Note that the apical frequency region does not cover the first formants of all vowels, and the middle and basal regions each cover the second formants of approximately half the vowels. The basal region also covers the third formant of all vowels (not shown).

The channels chosen for vocoder manipulation were based on analysis of the resonance frequencies of the vowel stimuli performed in Praat (Boersma and Weenink, 2013). The first, second, and third formants at the 25%, 50%, and 75% points of the duration of each vowel were identified from the estimated spectral peaks in each signal. This information was used to select low, middle, and high frequency channels (corresponding to apical, middle, and basal cochlear locations), so that the vocoder manipulations would affect vowel identification in predictable ways. Figure 1 shows the acoustic vowel spaces of the male and female talker vowels. Shaded areas indicate the low-, mid-, and high-frequency regions that were manipulated. Because the vowel space is different for female and male talkers and they are differentially affected by the manipulations, data from female and male talkers were analyzed separately.

**FIG. 2.**
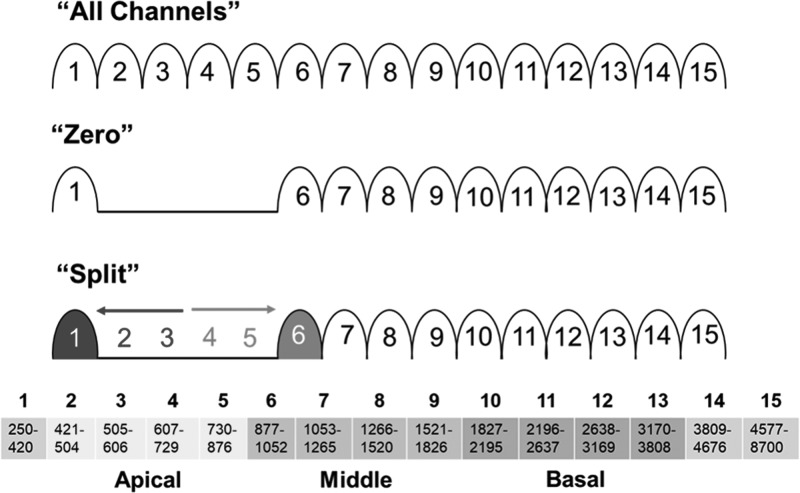
(Color online) Vocoder manipulations. The all channels control condition consisted of a 15-channel bandpass filter with no manipulations. In the zero condition, the output of particular channels was set to zero. In the split condition, particular channel output was also zero, but the frequency information from channels 2 and 3 (in this example), was sent through channel 1 in addition to the frequency components normally transmitted through channel 1. Similarly, the information from channels 4 and 5 is sent through channel 6, in addition to the normal frequency information transmitted through channel 6. The channels manipulated in this example are those that carry low-frequency information, corresponding to the apical region of the cochlea. Vocoder channel frequency allocations, corresponding to those of Advanced Bionics CIs, are shown at the bottom.

Spectral information was degraded via vocoder processing in one of two ways: (1) “zero”—the output of the filters of specified channels was set to zero, simulating complete loss of information through the channel, and (2) “split”—the output of specified filters was set to zero and half of the envelope energy from those filters were added to those of neighboring filters instead, simulating absence of information in the target channel as well as interaction with adjacent channels. Figure 2 illustrates these manipulations and channel frequency allocations.

The locations of the spectral manipulations were shifted through channels corresponding to apical (channels 2, 3, 4, and 5), middle (channels 6, 7, 8, and 9), and basal (channels 10, 11, 12, and 13) cochlear locations to vary the frequencies missing or altered from the speech spectrum. In addition, an “all channels” condition, in which stimuli underwent vocoder processing but were not further manipulated, served as a control condition. While the vocoder manipulations in the present study are similar in principle to those used by Shannon *et al*. (2002), this study used filter frequencies corresponding to those used by the clinical speech processors developed by Advanced Bionics Corp. (Valencia, CA). Thus the simulation of CI listening in the present study was more comparable to the clinical experience of Advanced Bionics CI users.

**FIG. 3.**
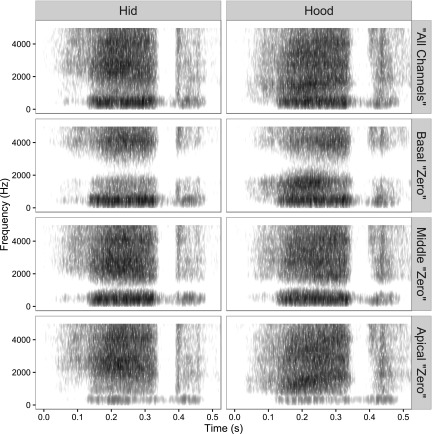
Spectrograms of vocoded vowels for the zero manipulation. Plotted as frequency (ordinate) over time (abscissa), with darker coloration indicating greater energy. Left column: Spectrograms for the vowel sound “hid” for the different regions of frequency removal. Right column: Spectrograms for the vowel sound “hood” for the different regions of frequency removal.

Figure 3 includes the spectrograms for female talker vowel stimuli “hid” (left column) and “hood” (right column) for the all channels condition (top), and each frequency region that was removed for the zero vocoder manipulation. “Hid” and “hood” have similar *F*1 but distinct *F*2 frequencies, evident in the all channels spectrogram. Frequencies corresponding to the *F*2 of “hood” were removed in the middle frequency region manipulation (third panel from the top) and the *F*2 frequencies of “hid” were removed in the basal frequency region manipulation (second panel from the top). In the absence of *F*2 cues, listeners relied more on *F*1 cues, and thus “hid” and “hood” were confused due to similarity of their *F*1 frequencies.

### D. Procedure

Testing was performed in a double-walled sound-treated booth (IAC RE-243). Stimuli were played through an external A/D device (SIIF USB SoundWave 7.1) and a Crown D75 amplifier and were presented at 60 dB-A through a Bose 161 speaker placed at 0° azimuth 1 m from the subject in the booth. Custom software (ListPlayer2 version 2.2.11.52, Advanced Bionics, Valencia, CA) was used to present the stimuli and record subject responses.

Male talker vowels, female talker vowels, and male talker consonants were presented in separate blocks. For each block, subjects completed two runs with three repetitions of each vowel or consonant with the vocoder manipulations pseudo-randomly interleaved, for a total of six data points for each speech token within each vocoder condition. After presentation of each sound, a list of possible choices was displayed on the computer screen and subjects used a computer mouse to select which one they thought they heard. Test runs were scored as percent correct and the two runs for each list were averaged for each subject. Confusion matrices for each list were also averaged across runs for each subject. Prior to testing a particular list, subjects completed a practice run consisting of one presentation of each vowel or consonant sound in the all channels condition only. Subjects could repeat the sound as many times as desired, and feedback was given after each response to familiarize participants with the vocoded stimuli and the task. Practice data were not included in the average performance scores or confusion matrices.

### E. Analysis

For each vocoder condition, phoneme identification scores were averaged across subjects for each list. Average male and female talker vowel and consonant recognition performance of each vocoder condition were compared to that of the control condition. One-way analysis of variance (ANOVA) with planned multiple comparisons was conducted to determine which conditions of degraded spectral information significantly decreased phoneme identification performance relative to the all channels condition.

In addition to statistical comparison of performance across conditions, the vowel and consonant sounds that were confused as a result of each vocoder condition were examined. Confusion matrix responses were pooled across subjects for each condition in order to conduct these analyses.

#### 1. SINFA

The amount of information related to vowel and consonant phonetic features transmitted by each vocoder condition was quantified using SINFA (Wang and Bilger, 1973). This analysis is based on that of Miller and Nicely (1955), and utilizes subjects' phoneme confusions to determine how different conditions of spectral degradation affect perception of phoneme features. However, since the contributions of each feature for phoneme recognition are not independent (e.g., as the nasal feature is recovered, the voicing feature is redundant, since all nasals are voiced), SINFA eliminates this internal redundancy. In the first iteration of the SINFA, the feature with the most perceptual importance for identification is selected and the percent of information transmitted for that feature is calculated. The effect of that feature is then held constant in the second iteration of the analysis, in which the feature with the second highest amount of perceptual information transmitted is identified and calculated, and so on. The analysis concludes when the contributions of all specified features have been elucidated.

To conduct the SINFA, vowels were categorized as having either short (<250 ms; lax vowels) or long (>250 ms; tense vowels) duration, low (<420 Hz), middle (420 to 520 Hz), or high (>520 Hz) *F*1 values (which correspond to high, mid and low vowels, respectively), and low (<1330 Hz), middle (1330 to 2000 Hz), or high (>2000 Hz) *F*2 values, consistent with the approach taken by Xu *et al.* (2005). Consonants were classified by their manner of articulation (stop, fricative, affricate, nasal, or liquid), place of articulation (bilabial, dental, alveolar, palatal, or velar) and voicing (voiced or unvoiced).

#### 2. Perceptual distance analysis

This analysis compares confusion matrices resulting from distinct conditions of spectral manipulation to evaluate the difference in phoneme perception between the two conditions (e.g., Zaar and Dau, 2015). Each cell of one confusion matrix is compared to the corresponding cell in the other confusion matrix to determine the overall difference in phoneme perception between the two matrices, scaled from 0% to 100%: a distance of 0% indicates that the matrices compared are exactly the same, and a distance of 100% indicates complete dissimilarity between the matrices. Baseline perceptual distance for the analyzed matrices is determined by calculating the within-subject perceptual distance. For example, Zaar and Dau (2015) applied this process to consonant identification confusion matrices to determine the effects of different talkers, types of noise used, and listeners on consonant perception. They calculated the perceptual distance within each subject from test and retest runs and used the resulting values as a baseline for other measurements, since these values represent listener uncertainty. Therefore, this study also calculated a baseline by examining the perceptual distance between each subject's confusion matrices from the first and second runs from each vocoder condition for each phoneme list. Results from perceptual distance calculations were compared to these baseline values, and meaningful results were determined to be those that were significantly higher than the calculated baseline values. In this study, perceptual distance was calculated between listeners for the “all channel” condition to determine the variability among subjects' responses in the control condition. Perceptual distance was also calculated between the male and female talker vowels for the all channels condition to determine the effect of speaker on vowel perception. In addition, this analysis was used to compare the zero and split manipulations for each degraded frequency region within each phoneme list to obtain the differences in phoneme perception due to vocoder manipulation. These calculations were performed within each subject and then averaged across subjects.

#### 3. Perceptual vowel space analysis

The goal of the perceptual vowel space analysis was to determine the direction of the errors resulting from each vocoder manipulation, in terms of physical articulator space and acoustic space. This analysis demonstrates the tendency of specific channel manipulations to warp the perceptual vowel space in a way that can be illustrated with a traditional two-dimentional vowel map. Each vowel was assigned a feature value between 1 and 5 for height (1 = low, 5 = high) and for advancement (1 = back, 5 = front). These numbers reflect the general phonological feature distribution of the vowel space rather than exact formant frequencies, which cannot be easily resolved down to single numbers on account of their dynamically changing state. The difference in feature values between each target and responded vowel was calculated in order to translate confusion matrices into summaries of vowel feature perception and to aid in the illustration of modified perceptual vowel spaces.

### F. Comparisons with cochlear implant listeners

The present study was a simulation of suboptimal CI electrode-neuron interfaces and accordingly, the confusion patterns made by NH listeners were compared to those made by CI users. Two CI listeners in particular were selected because they were judged in a previous study (DeVries *et al*., 2016) to have poor electrode-neuron interfaces in only the middle frequency region (S43) or the middle and basal frequency regions (S47), somewhat matching those used in this study.

DeVries *et al*. (2016) measured auditory perception thresholds with focused electrical stimulation as a method to identify the potential locations of suboptimal electrode-neuron interfaces. These thresholds were obtained using a steered quadrupolar electrode configuration, which consists of four intracochlear electrodes: two middle electrodes serve as active electrodes, and two outer electrodes serve as return electrodes. Stimuli consisted of biphasic, charge-balanced pulse trains with a 102 *μ*s phase duration and pulse rate of 997.9 presented directly to the CI through the Bionic Ear Data Collection System version 1.18.315 (Advanced Bionics, Valencia, CA). Participants performed an adaptive two-up one-down, two-interval forced choice procedure in which they identified the interval that contained the sound. Each run contained six reversals. For the first two reversals, step size increased by 2 dB for correct responses or decreased 2 dB for incorrect responses. The step size decreased to 0.5 dB for the remaining four reversals. Each reversal converged at 70.7% correct on the psychometric function (Levitt, 1971). Thresholds were determined based on the average of the last four reversals. Four runs were collected and averaged for each electrode. The procedure was repeated until focused auditory perception thresholds were obtained for electrodes 2 to 15.

## III. RESULTS

### A. Vowel identification performance

**FIG. 4.**
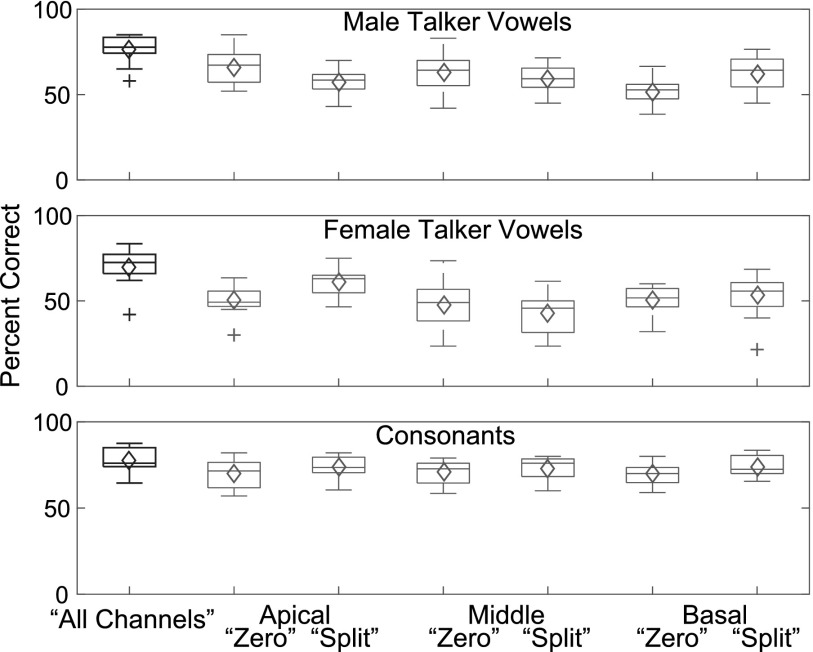
(Color online) Vowel and consonant identification performance. Box plots depict the average performance across subjects for each condition for each phoneme list. Lower and upper end of the boxes indicate the 25th and 75th percentiles, respectively. Whiskers extend from the third quartile to the highest value that is +1.5 × the interquartile range and from the first quartile to the lowest value that is −1.5 × the interquartile range. Plus signs indicate outliers. The middle line of each boxplot is the median and the diamond symbols represent the mean.

Significant differences were found in average phoneme identification performance between the seven vocoder conditions for male talker vowels [*F*(6, 77) = 8.64, *p* < 0.001] and female talker vowels [*F*(6, 77) = 8.03, *p* < 0.001], but not for consonants [*F*(6, 77) = 2.07, *p* = 0.067]. For both vowel lists, planned multiple comparisons indicated that all combinations of vocoder manipulation and location resulted in significantly lowered identification compared to the all channels condition at an alpha level of 0.05. However, when a Bonferroni correction was applied to correct for multiple comparisons (0.05/18, α = 0.002), the vowel identification scores for the female talker Apical zero condition and the male talker Apical split condition were no longer significantly lower than the all channels condition. The results for all other conditions for vowels remained significant. For consonants, neither the zero nor the split manipulations significantly lowered consonant identification (α = 0.002) relative to the control condition for any frequency region, although the split manipulation resulted in slightly better identification performance compared to the zero manipulation. Table I depicts the *p* values for each planned comparison from the ANOVA performed for each phoneme list. Figure 4 illustrates the median and average phoneme identification performance for each vocoder condition within a list.

**TABLE I.** The *p* values resulting from comparisons between each vocoder condition and the all channels control condition. Shaded cells indicate a significant result at the *p* < 0.002 level.

	Apical Zero	Middle Zero	Basal Zero	Apical Split	Middle Split	Basal Split
Female talker vowels	0.006	<0.001	<0.001	<0.001	<0.001	<0.001
Male talker vowels	<0.001	<0.001	<0.001	0.038	<0.001	<0.001
Consonants	0.008	0.013	0.005	0.144	0.092	0.191

### B. Phoneme confusions

**FIG. 5.**
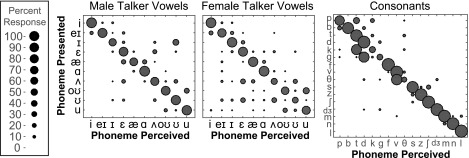
(Color online) Confusion matrices for the all channels control condition averaged across subjects. Responses are plotted as phoneme presented (ordinate) vs phoneme perceived (abscissa). The size of the circle indicates the percentage of response.

Responses from the all channels condition indicated that vowel and consonant confusions were made even when spectral channels were not dropped or reallocated, probably because the spectral resolution of the all channels condition was still limited compared to natural speech (Fig. 5). For example, listeners made identification errors in this condition for the male talker vowels in the words “hid,” “had,” “hud,” and “hood,” and for the female talker vowels in the words “who'd,” “hood,” “hoed,” and “hud,” and the consonants in “aGa,” “aKa,” and “aTHa.” However, between-subject perceptual distance for each phoneme in the all channels condition (29.6% for male talker vowels, 32.8% for female talker vowels, and 17.7% for consonants) were comparable to baseline within-subject perceptual distance values for this condition, indicating that overall limited spectral resolution resulted in similar phoneme error patterns for all subjects.

**FIG. 6.**
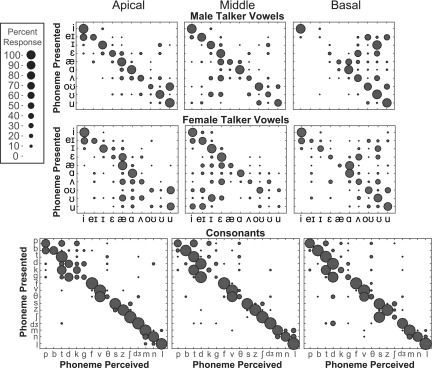
(Color online) Confusion matrices for the zero vocoder manipulation for each frequency region. Responses are plotted as phoneme presented (ordinate) vs phoneme perceived (abscissa). Percent responded is indicated by circle size, such that larger circles indicate higher percentage of response. Vowels are ordered according to vowel space (see Fig. 1), starting with “heed” and moving counterclockwise. Adjacent vowels have similar second formants and vowels opposite each other have similar first formants. Consonants are ordered by manner of articulation (stops, fricatives, affricates, nasals, and liquid).

Patterns of vowel confusions from the vocoder conditions reflected the region of degraded information. Figure 6 shows the male and female talker vowel and consonant confusion matrices for the zero vocoder manipulation. Confusion patterns were very similar between the zero and split conditions. In these plots, adjacent vowels have similar second formant values and vowels on opposite ends of the matrix have similar first formant values. Manipulation of frequency regions corresponding to first formants (apical regions) resulted in confusions of vowels with similar second formants (i.e., similar vowel advancement). Manipulation of frequency regions corresponding to second formants (middle regions for back vowels and basal regions for front vowels) resulted in confusions between vowels with similar first formants (i.e., similar vowel height). In Fig. 6, consonants are ordered by manner of articulation. Consonant confusions occurred between those most similar in manner, exhibited by confusions clustered around the diagonal, and are generally consistent for all conditions of spectral manipulation.

**FIG. 7.**
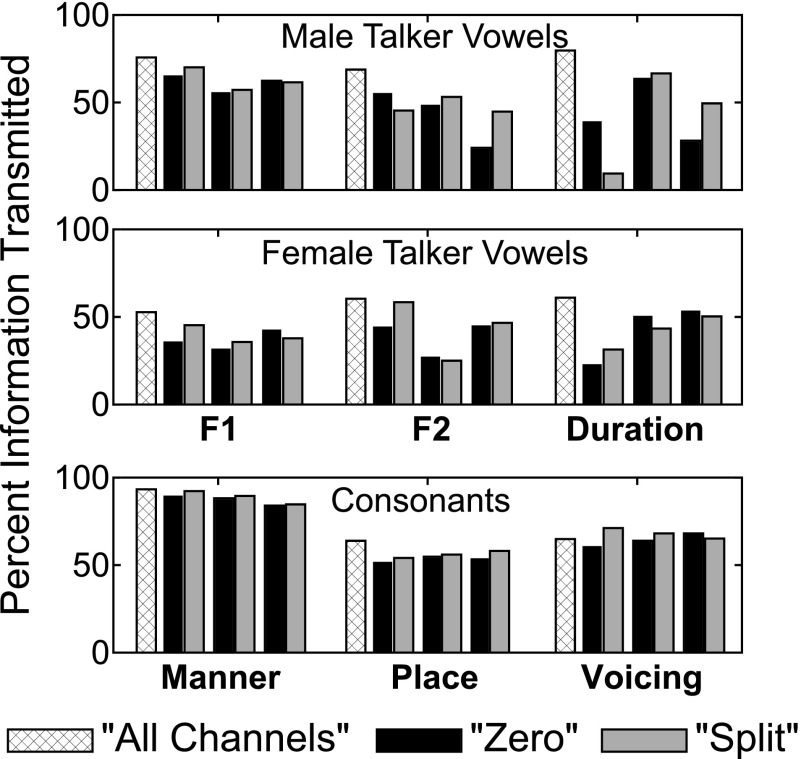
(Color online) Results of SINFA for male and female talker vowels and consonants. Bars are grouped for each investigated phonetic feature by the apical, middle, and basal regions. The height of each bar depicts the percent of information transmitted for that feature within the specified condition.

Figure 7 shows the results of the SINFA for each condition for male and female talker vowels and consonants. Percent of information transmitted in the all channels condition for all phoneme lists was greater or not significantly different from information transmitted in other vocoder conditions. For vowels, no clear patterns were found for the amount of information transmitted between vocoder manipulation types or locations, perhaps because of the lack of independence between acoustic attributes of vowels in English. However, SINFA results for consonants revealed a high amount of information transmitted for manner in all conditions, consistent with the relatively intact temporal envelope transmitted through a vocoder, which would yield cues for manner. The split condition transmitted slightly more information related to manner than did the zero conditions, but consonant feature transmission overall was much less affected by particular vocoder manipulations than vowels were. These results are consistent with identification performance results and also in agreement with the acoustic cues available for each type of sound category.

**FIG. 8.**
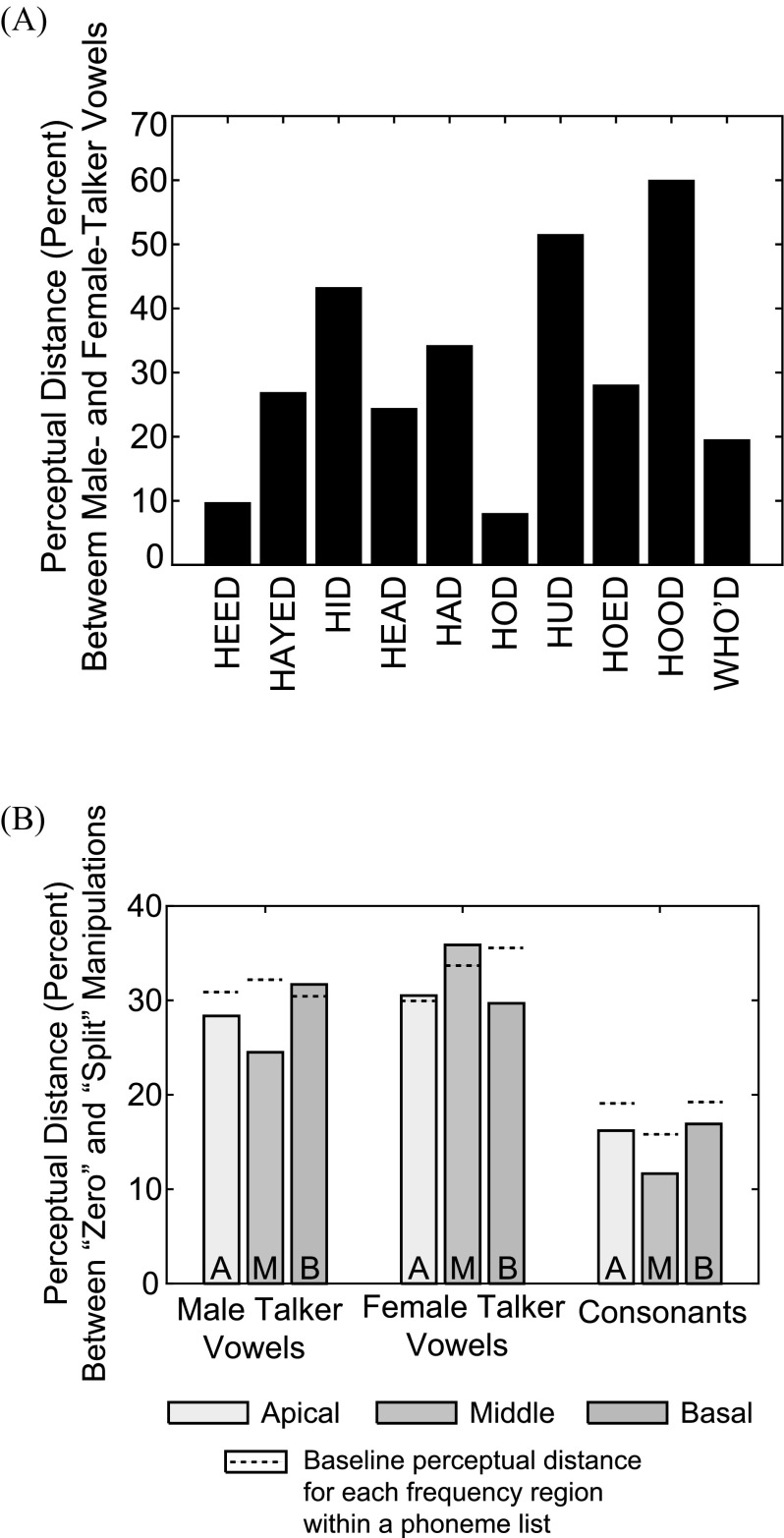
(Color online) Perceptual distance results. (A) Male and female talker vowels. Results shown are from the all channels control condition and were averaged across subjects. Each bar represents one vowel and the height of the bar indicates the perceptual distance for that vowel between talkers, in percent. (B) Zero and split conditions. Results were averaged across phonemes within a list and subsequently across subjects. Bars are grouped by phoneme list. Bar height indicates the perceptual distance in percent between the two vocoder manipulation types. The dashed line within or above each bar indicates the baseline within-subject perceptual distance between the zero and split manipulations for that particular frequency location and phoneme list.

The perceptual distance values between male and female talker vowels for the all channels condition depended on the phoneme being examined [see Fig. 8(A)]. Responses to the cardinal vowels (/i/, /ɑ/, and /u/) were similar between male and female talkers resulting in perceptual distance values (7.9%, 9.6%, and 19.4%, respectively) that were smaller than the within-subject baseline perceptual distance (24.1%) that was calculated between male and female speakers. Responses to other, more centralized vowels were quite variable between the two speakers resulting in larger perceptual distance values (24.3%-59.9%) and greater magnitude of perceptual distance than the baseline value of 24.1%.

Figure 8(B) shows the perceptual distance calculations between zero and split for the apical, middle, and basal frequency regions. These values are either lower than or not significantly higher than the baseline perceptual distance for each frequency region within a phoneme list, indicating non-significant differences in perception due to vocoder manipulation type.

**FIG. 9.**
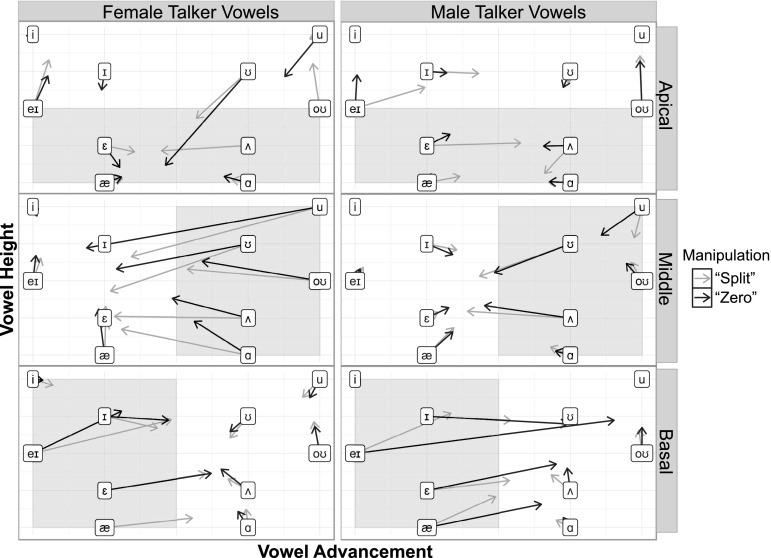
(Color online) Acoustic vs perceived vowel space. Placement of vowel symbols correspond to their height and advancement in acoustic vowel space. Gray shaded areas indicate the region of vowel space that was manipulated for the apical, middle, and basal vocoder locations. Arrows depict the shift in vowel perception due to vocoder manipulations in particular frequency regions.

The reliable arrangement of vowels in a two-dimensional acoustic space enables visualization of how perceptions can drift from one acoustic region to another. For most vowels, perceptual vowel space was shifted when spectral information was missing or distorted. Figure 9 shows the shifts in perceptual vowel space resulting from specific vocoder manipulations. The apical vocoder conditions in which the low-frequency (apical) region, typically containing the first formant, was manipulated resulted in some shifts in perceived vowel height (indicated by arrows pointing upward or downward). Errors in perceived vowel advancement typically indicated that perceptions shifted *away* from the area of spectral distortion. Similarly, for the vocoder conditions that manipulated the middle frequencies corresponding to back vowels' low second formants, more “front” vowels were perceived (arrows pointing leftward). For manipulations of the higher-frequency basal regions, front vowels with high second formants were perceived as being more “back” (indicated by arrows pointing rightward). It is notable that these errors are not symmetrical; vowel pairs were not confusable; perception of specific vowels shifted toward other specific vowels.

**FIG. 10.**
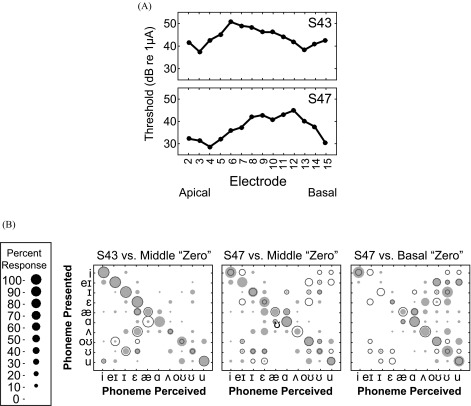
(Color online) Comparison between NH and CI listener vowel confusions. (A) Focused threshold profiles for two CI subjects, S43 and S47. S43 exhibits elevated thresholds, indicating suboptimal electrode-neuron interfaces, in the channels corresponding to the middle vocoder frequency region. S47 has elevated focused thresholds in channels corresponding to both the middle and basal vocoder frequency regions. (B) Vowel confusion matrices from CI subjects S43 and S47 (colored circles) overlaid on average NH listener data (gray filled circles).

Comparison of consonant and vowel confusions between NH listeners in the vocoder simulation and the CI users tested revealed similar confusion patterns between the groups. For NH and CI listeners, /p/ was confused with /t/ and /k/, /g/ was confused with /d/, /n/ was confused with /m/, and /k/ was confused with /t/ and /p/. These confusions were unidirectional, analogous to patterns of vowel confusions. These are also classic consonant confusions resulting from other kinds of signal degradation (Miller and Nicely, 1955). Perceptual distance analysis results indicated that vowel confusion patterns made by each CI listener were somewhat analogous to the confusions made by NH listeners in the vocoder condition(s) that best matched the CI users' region of elevated focused thresholds (which likely indicate locations of suboptimal electrode-neuron interfaces). Figure 10(A) shows the focused threshold profiles of the two example CI users. These listeners exhibit elevated focused thresholds in the channels corresponding to the middle (S43) and middle and basal (S47) frequency regions used in the vocoder experiments. Figure 10(B) shows that S43 made vowel confusions comparable to those made by NH listeners in the middle zero vocoder condition. S47 had two regions of elevated thresholds that were manipulated separately in the present study, and Fig. 10(B) shows that this subject also made confusions somewhat similar to NH listeners in both the middle and basal zero vocoder conditions. Table II contains the perceptual distance values that compare these CI listeners' male and female talker vowel identification confusion matrices to those of NH listeners from all vocoder conditions. The lowest perceptual distance values, indicating the most similar confusion patterns, were observed between S43's confusion matrices and the middle zero and split vocoder conditions. The perceptual distances between S47's confusion matrix and middle and basal vocoder conditions are greater than they might have been if the present study had used a larger span of manipulated channels to match this listener.

**TABLE II.** Perceptual distance between CI subjects S43 and S47 and NH listeners' vowel identification confusion matrices for each vocoder condition. The smallest perceptual distance values, which signify the most similar confusion patterns, are between S43's confusion matrices and the vocoder conditions that manipulated the middle frequency regions.

Subject Number	Versus	Apical		Middle		Basal	
		Zero	Split	Zero	Split	Zero	Split
S43	Male talker	49.83	58.13	36.77	34.11	73.14	57.92
	Female talker	56.01	49.09	39.83	36.44	66.02	62.52
S47	Male talker	52.55	55.12	51.91	55.86	48.32	49.24
	Female talker	48.20	37.10	45.58	54.52	46.16	40.93

## IV. DISCUSSION

In this study, NH listeners participated in a simulation of CI listening in which vowels and consonants were spectrally degraded to mimic the effects of localized suboptimal electrode-neuron interfaces on phoneme perception. Systematic analyses were performed on the resulting phoneme identification scores and confusion patterns.

Results of phoneme identification performance from the present study indicated that both the zero and split manipulations significantly lowered vowel identification relative to the all channels condition. Only small differences, however, were found between the effects of the zero and split manipulations on vowel recognition performance and confusion patterns in the present study. These findings indicate that distortion of the frequency spectrum is as detrimental to vowel identification as complete loss of particular frequency ranges. This pattern did not hold true for consonant identification, which was not significantly reduced in the zero or split manipulations compared to the all channels condition. These results suggest that consonant perception is robust to frequency distortion.

### A. Relation to previous studies

A comparison of the current results with those of Shannon *et al*. (2002) shows consistency in the finding that the zero and split conditions deteriorate performance for vowels. However, Shannon *et al*. (2002) found that both manipulations significantly decreased consonant recognition scores, while the current study showed that consonant intelligibility was not significantly affected by the zero or split conditions. This discrepancy may be the result of differences in stimuli, and in frequency band allocation. Shannon *et al*. (2002) based their vocoder processing on the Cochlear Corporation SPEAK CI processor and used 20 filter bands with analysis filters from 150 to 10 823 Hz. In contrast, the present study modeled vocoder processing after Advanced Bionics Fidelity 120 CI processing and used both a smaller number of filter bands (15) and a smaller range of frequency analysis filters (250 to 8700 Hz) and a different frequency allocation table. Accordingly, the spectral “holes” used by Shannon *et al*. (2002) ranged from 1.1 mm (two channels in the basal location) to 10.7 mm (eight channels in the apical location) of cochlear space, while those used in the present study were 4.3, 4.8, and 5.0 mm for the four channels whose output was manipulated in the apical, middle, and basal frequency regions, respectively (Greenwood, 1990).

Performance for both vowels and consonants was below ceiling even for the all channels condition, demonstrating the difficulty of phoneme perception when spectral resolution is degraded. In a previous vocoder experiment, Litvak *et al*. (2007) tested NH listeners on vowel and consonant identification with 5, 10, 20, or 40 dB/octave output filter slopes (with higher numbers corresponding to progressively better resolution) and found that shallower filter slopes, indicated by lower dB/octave values, resulted in decreased phoneme identification scores compared to narrower filter slopes. The present study used the same type of vocoder processing and a 30 dB/octave output filter slope, which in pilot testing was the slope that resulted in comparable phoneme identification performance between NH listeners participating in this experiment and the better performing CI listeners who identified natural stimuli in a previous study (DeVries *et al.*, 2016). Results from the present study are consistent with findings from Litvak *et al*., in that NH listeners' average vowel and consonant identification scores from the all channels condition with the 30 dB/octave filter slope fall between the range of performance that Litvak *et al*. observed with their 20 dB/ octave and 40 dB/octave filter slopes.

In this study, the hypothesis was tested that manipulations of particular formant frequencies would lead to predictable vowel identification errors, i.e., those vowels whose formants are removed or distorted for each condition will have the most errors in that condition. Examination of vowel confusion patterns indicates that vowel errors are indeed predicted based on the frequency information that is degraded. Furthermore, errors were asymmetrical, tending to gravitate away from the area of manipulation and into the area of relative spectral preservation. In conditions that manipulated the frequencies corresponding to the first formant of a vowel, confusions of vowel height were made, and vowels were confused with other vowels that have similar second formants, indicating the preservation and reliance on second formant information in the absence of first formant cues. While the same pattern was observed in the conditions in which second formant frequency information was degraded, errors on perception of vowel advancement were made to a higher degree than corresponding degradations of vowel height. Again, confusions of vowels according to *F*2 resulted in perceptions of vowels with similar *F*1, indicating correct perception of at least one feature rather than complete misperception. The first formant was more robust to degradation than the second formant, perhaps because the Advanced Bionics electrode array, which this vocoder study simulated, has more space devoted to transmitting *F*1 frequencies (six channels) than *F*2 frequencies (four channels).

Despite the different vocoder manipulations and locations of manipulated frequency regions, some vowels seemed to be inherently easier to identify than others. For example, for both male and female talker lists, “heed” was identified from 84% to 98% correct for all vocoder conditions. Conversely, “hud” was often misidentified independent of vocoder manipulation or location. It could be the case that cardinal vowels, having fewer acoustical neighbors, are more robust to degradation, while lax vowels have a higher number of potential confusable pairs.

Results of consonant error pattern analyses indicated that confusions occurred between consonants with the most similar manner of articulation, regardless of the vocoder condition. These results corroborate the idea of the vocoder as preserving temporal envelope structure (a key determiner of manner of articulation) and coincide with results from the vocoder stimulation in NH listeners conducted by Kasturi *et al*. (2002). They performed perceptual weighting of the vocoder channels to determine the region(s) of frequency information most critical for vowel and consonant identification. While they found that some channels were weighted higher than others for vowel recognition, the weighting function for consonant identification was flat. This indicated that all frequency regions contributed equally to consonant recognition, or that there was greater interdependence of frequency channels for consonants. In the present study, as indicated by consonant confusion patterns, NH listeners were able to use temporal cues reliably to accurately identify consonant manner, even under conditions of spectral degradation. This resulted in patterns of consonant errors that preserved some feature(s) of the target sound, rather than complete misperceptions. These findings support the prediction that vowel stimuli are more useful than consonants in studies that intend to measure effects of frequency-specific degradations. Since adequate spectral resolution is more important for vowel recognition than consonant recognition, use of vowel stimuli can provide valuable information about the perceptual effects of distorted frequency regions.

The SINFA results from the current study demonstrated that the all channels vocoder condition transmitted the largest amount of information compared to the other conditions for both vowels and consonants, validating the spirit of the measurement and corroborating phoneme identification performance. For consonants, SINFA findings were consistent with confusion patterns, indicating that manner of articulation was the feature with the highest percentage of information transmitted. These results correspond to the preservation of temporal aspects of the manner feature during vocoder processing. Place of articulation exhibited the lowest information transmitted, due to the dominance of the spectral information that was degraded. This finding is in line with results from previous studies that tested consonant recognition under conditions of spectral degradation (for example, Dorman *et al.*, 1997; Xu *et al.*, 2005).

While the SINFA yielded clear patterns of results relating to feature perception of spectrally manipulated consonants, this analysis failed to elucidate changes in vowel feature perception due to manipulated frequency regions or type of vocoder manipulation. Results of the SINFA should have coincided with patterns of errors on vowel perception (for example, vowel height errors when apical regions were distorted). This shortcoming is likely because of the relative dependence of features for vowels. A fundamental assumption of SINFA (or any information-theoretic approach to confusion patterns) is that phoneme features are independent bits of information. This independence can be argued for consonants: e.g., alveolar consonants should have prototypical formant trajectories regardless of manner of articulation, and stop sounds should have a particular temporal pattern regardless of place of articulation, although consonants do have unequal numbers of connections between features which violate the information-theoretic assumptions of SINFA. On the other hand, acoustic properties of vowels, such as *F*1 and *F*2, are decidedly interdependent (Syrdal and Gopal, 1986). For example, front vowels with lower *F*1 will also have higher *F*2, and longer vowels will generally have higher *F*1, though this is affected considerably by tense/lax status. In short, there is no example in the English vowel system where only one acoustic parameter is changed independently of the others. Therefore, while SINFA may be satisfactory for analysis of consonant identification confusions, other analysis methods, such as the perceptual distance or vowel space analysis, can be more appropriate for examination of vowel perception confusion patterns.

The perceptual vowel space analysis was the most informative for the purposes of this study. These results showed how each condition of spectral degradation warped perception of vowels in articulatory-acoustic space. Shifts in perception of vowel height and advancement were clearly related to the spectral information manipulated in each condition. In the conditions in which frequencies corresponding to vowels' first formants were manipulated (apical region), some vowels shifted in perceived height. In conditions where low values of *F*2 were manipulated (the “middle” frequency region), perception drifted toward more front vowels, and vice versa for front vowels whose *F*2 was manipulated with basal frequency degradation.

The findings from these experiments and other vocoder studies (for example, Shannon *et al.*, 2002; Litvak *et al.*, 2007) show similar decreases in speech identification performance between CI users and NH listeners under certain conditions of spectral degradation. Still, a study of how CI users' phoneme confusion patterns relate to those of NH listeners identifying spectrally degraded stimuli had not yet been examined. Comparison of confusion patterns from NH listeners in this study and CI listeners revealed similar patterns of consonant errors. Even more interesting, however, was that the spectral manipulations in the present study resulted in NH listener vowel confusions that were akin to those made by CI users with poor electrode-neuron interfaces in similar frequency regions as those in the present simulation study. While S43's region of elevated focused thresholds matched one region of frequency manipulation used in the present study (middle), S47's elevated focused thresholds spanned two of the frequency regions manipulated in this experiment (middle and basal). Thus, S43's vowel confusion patterns were more similar to those made by NH listeners in the middle vocoder conditions than were S47's vowel confusions compared to the middle and basal vocoder conditions. If the present study had included a vocoder condition with a larger frequency range that matched the entire region of S47's elevated focused thresholds, it is likely that NH listeners' vowel confusion patterns would be more comparable to those of S47. These findings suggest that simulations of spectral holes are reasonable for mimicking the poor interfaces between CI electrodes and spiral ganglion neurons.

Because of the significance of spectral cues for accurate vowel identification exemplified by this study and others, analysis of the vowel confusions of CI users may allow for the prediction of CI subject-specific causes of poor spectral cue transmission. For instance, Harnsberger *et al*. (2001) mapped the perceptual vowel spaces of CI listeners resulting from basalward frequency shifts, and while they found that most CI users tested were able to adapt to the frequency shift, they observed differences in perceptual vowel space between NH listeners and individual CI users in terms of acoustic space and compactness of vowel categories. Therefore, future work could involve perceptual vowel space analysis of the vowel identification confusion matrices of CI listeners for whom the locations of poor electrode-neuron interfaces have been predicted. Such analyses could identify the particular spectral cues that are missing due to the effects of suboptimal electrode-neuron interfaces. CI users' speech processor settings or stimulation modes could then be changed in an attempt to restore the missing spectral information.

Previous studies have shown that experimental CI processor programs can improve CI user speech identification performance. Zwolan *et al*. (1997) found that some CI users exhibited higher scores on sentences, monosyllabic words, and phonemes within monosyllabic words while using experimental maps that contained only the electrodes they could discriminate on a previous task. Similar investigations have demonstrated that CI user speech perception can be improved by deactivating channels determined to be suboptimal based on particular criteria, and reallocating those frequencies to active electrodes. For example, deactivating channels with a high level of forward masking (Boëx *et al*., 2003), poor temporal sensitivity (Garadat *et al*., 2013), or channels for which a computational model predicted a high degree of interaction with other electrodes (Noble *et al.*, 2013) and reallocating frequencies to active electrodes have been found to result in better CI user performance on tests of consonant identification in quiet (Boëx *et al.*, 2003; Garadat *et al.*, 2013), sentence recognition in noise (Garadat *et al.*, 2013), and performance on the Bamford-Kowal-Bench Speech-In-Noise (BKB-SIN) test (Noble *et al.*, 2013). Similarly, Bierer and Litvak (2016) found that deactivating channels with high focused thresholds, thereby eliminating channels with suboptimal electrode-neuron interfaces, and reallocating frequencies to remaining electrodes increased consonant and vowel identification scores for some individuals. Larger improvements for a greater number of CI users may be observed if channels selected for deactivation were more targeted: such channels would be determined to have poor-electrode neuron interfaces but also be affecting perception of particular vowels, based on perception of stimuli with frequency-specific cues. In this study, we showed that vowels, in account of their arguably well-defined acoustic structure, could provide a good probe for frequency-specific deficits in a way that word or sentence stimuli cannot target as specifically.

## V. CONCLUSIONS

This study simulated the spectrally degrading effects of suboptimal CI electrode-neuron interfaces on vowel and consonant recognition of NH listeners. As predicted, vowel identification performance significantly decreased with a loss or distortion of frequency information, but consonant recognition was less affected by the type of distortion, consistent with previous studies. This study utilized relatively novel techniques, perceptual distance and vowel space analyses, to examine phoneme confusion patterns. Results indicated that vowel confusions occurred between those most similar in residual frequency space and consonant confusions occurred between those with the same manner of articulation. Perception of vowels specifically *drifted away* from areas of frequency distortion, rather than simply causing uncertainty and greater number of errors. Vocoder spectral degradation in NH listeners resulted in patterns of vowel and consonant errors that are similar to those made by example CI users with matching region(s) of suboptimal electrode-neuron interfaces. This is the first experiment to demonstrate that perception will drift away from areas of frequency-specific distortion in a predictable manner. These results provide insight into the perceptual consequences of spectral distortion on speech confusions. These findings may be useful for interpreting speech confusions of individual CI users and translating those data into diagnostic markers of the electrode-neuron interface.

## References

[1] Ainsworth, W. A. (1972). “ Duration as a cue in the recognition of synthetic vowels,” J. Acoust. Soc. Am. 51, 648–651. 10.1121/1.1912889

[2] Assmann, P. F. , and Katz, W. F. (2005). “ Synthesis fidelity and time-varying spectral change in vowels,” J. Acoust. Soc. Am. 117, 886–895. 10.1121/1.1852549 15759708

[3] Bierer, J. A. (2007). “ Threshold and channel interaction in cochlear implant users: Evaluation of the tripolar electrode configuration,” J. Acoust. Soc. Am. 121, 1642–1653. 10.1121/1.2436712 17407901

[4] Bierer, J. A. (2010). “ Probing the electrode-neuron interface with focused cochlear implant stimulation,” Trends Amplif. 14, 84–95 10.1177/1084713810375249. 20724356 PMC4111350

[5] Bierer, J. A. , and Faulkner, K. F. (2010). “ Identifying cochlear implant channels with poor electrode-neuron interface: Partial tripolar, single-channel thresholds and psychophysical tuning curves,” Ear Hear. 31, 247–258. 10.1097/AUD.0b013e3181c7daf4 20090533 PMC2836401

[6] Bierer, J. A. , and Litvak, L. (2016). “ Reducing channel interaction through cochlear implant programming may improve speech perception: Current focusing and channel deactivation,” Trends Hear. 20, 1–12. 10.1177/2331216516653389 PMC4948253 27317668

[7] Bingabr, M. , Espinoza-Varas, B. , and Loizou, P. C. (2008). “ Simulating the effect of spread of excitation in cochlear implants,” Hear Res. 241, 73–79. 10.1016/j.heares.2008.04.012 18556160 PMC2596864

[8] Boersma, P. , and Weenink, D. (2013). “ Praat: Doing phonetics by computer (version 5.3.56),” from http://www.praat.org (Last viewed January 8, 2014).

[9] Boëx, C. , Kós, M.-I. , and Pelizzone, M. (2003). “ Forward masking in different cochlear implant systems,” J. Acoust. Soc. Am. 114, 2058–2065. 10.1121/1.1610452 14587605

[10] DeVries, L. , Scheperle, R. , and Bierer, J. A. (2016). “ Assessing the electrode-neuron interface with the electrically evoked compound action potential, electrode position, and behavioral thresholds,” J. Assoc. Res. Otolaryngol. 17, 237–252. 10.1007/s10162-016-0557-9 26926152 PMC4854826

[11] Dorman, M. F. , Hannley, M. T. , Dankowski, K. , Smith, L. , and McCandless, G. (1989). “ Word recognition by 50 patients fitted with the Symbion multichannel cochlear implant,” Ear Hear. 10, 44–49. 10.1097/00003446-198902000-00008 2721828

[12] Dorman, M. F. , Loizou, P. C. , and Rainey, D. (1997). “ Speech intelligibility as a function of the number of channels of stimulation for signal processors using sine-wave and noise-band outputs,” J. Acoust. Soc. Am. 102, 2403–2411. 10.1121/1.419603 9348698

[13] Dudley, H. (1939). “ The automatic synthesis of speech,” Proc. Natl. Acad. Sci. U.S.A. 25, 377–383. 10.1073/pnas.25.7.377 16577919 PMC1077925

[14] Finley, C. C. , Holden, T. A. , Holden, L. K. , Whiting, B. R. , Chole, R. A. , Neely, G. J. , Hullar, T. E. , and Skinner, M. W. (2008). “ Role of electrode placement as a contributor to variability in cochlear implant outcomes,” Otol. Neurotol. 29, 920–928. 10.1097/MAO.0b013e318184f492 18667935 PMC2663852

[15] Firszt, J. B. , Holden, L. K. , Skinner, M. W. , Tobey, E. A. , Peterson, A. , Gaggl, W. , Runge-Samuelson, C. L. , and Wackym, P. A. (2004). “ Recognition of speech presented at soft to loud levels by adult cochlear implant recipients of three cochlear implant systems,” Ear Hear. 25, 375–387. 10.1097/01.AUD.0000134552.22205.EE 15292777

[16] Friesen, L. M. , Shannon, R. V. , Başkent, D. , and Wang, X. (2001). “ Speech recognition in noise as a function of the number of spectral channels: Comparison of acoustic hearing and cochlear implants,” J. Acoust. Soc. Am. 110, 1150–1163. 10.1121/1.1381538 11519582

[17] Fu, Q. J. , and Shannon, R. V. (1999). “ Recognition of spectrally degraded and frequency-shifted vowels in acoustic and electric hearing,” J. Acoust. Soc. Am. 105, 1889–1900. 10.1121/1.426725 10089611

[18] Garadat, S. N. , Zwolan, T. A. , and Pfingst, B. E. (2013). “ Using temporal modulation sensitivity to select stimulation sites for processor MAPs in cochlear implant listeners,” Audiol. Neurootol. 18, 247–260. 10.1159/000351302 23881208 PMC3874548

[19] Gifford, R. H. , Shallop, J. K. , and Peterson, A. M. (2008). “ Speech recognition materials and ceiling effects: Considerations for cochlear implant programs,” Audiol. Neurootol. 13, 193–205. 10.1159/000113510 18212519

[20] Greenwood, D. D. (1990). “ A cochlear frequency-position function for several species–29 years later,” J. Acoust. Soc. Am. 87, 2592–2605. 10.1121/1.399052 2373794

[21] Harnsberger, J. D. , Svirsky, M. A. , Kaiser, A. R. , Pisoni, D. B. , Wright, R. , and Meyer, T. A. (2001). “ Perceptual ‘vowel spaces' of cochlear implant users: Implications for the study of auditory adaptation to spectral shift,” J. Acoust. Soc. Am. 109, 2135–2145. 10.1121/1.1350403 11386565 PMC3433712

[22] Henry, B. A. , Turner, C. W. , and Behrens, A. (2005). “ Spectral peak resolution and speech recognition in quiet: Normal hearing, hearing impaired, and cochlear implant listeners,” J. Acoust. Soc. Am. 118, 1111–1121. 10.1121/1.1944567 16158665

[23] Hillenbrand, J. , Getty, L. A. , Clark, M. J. , and Wheeler, K. (1995). “ Acoustic characteristics of American English vowels,” J. Acoust. Soc. Am. 97, 3099–3111. 10.1121/1.411872 7759650

[24] Hillenbrand, J. M. , and Nearey, T. M. (1999). “ Identification of resynthesized /hVd/ utterances: Effects of formant contour,” J. Acoust. Soc. Am. 105, 3509–3523. 10.1121/1.424676 10380673

[25] Hirata, Y. , and Tsukada, K. (2009). “ Effects of speaking rate and vowel length on formant frequency displacement in Japanese,” Phonetica 66, 129–149. 10.1159/000235657 19776664

[26] Holden, L. K. , Finley, C. C. , Firszt, J. B. , Holden, T. A. , Brenner, C. , Potts, L. G. , Gotter, B. D. , Vanderhoof, S. S. , Mispagel, K. , Heydebrand, G. , and Skinner, M. W. (2013). “ Factors affecting open-set word recognition in adults with cochlear implants,” Ear Hear. 34, 342–360. 10.1097/AUD.0b013e3182741aa7 23348845 PMC3636188

[28] Jolly, C. N. , Spelman, F. A. , and Clopton, B. M. (1996). “ Quadrupolar stimulation for cochlear prostheses: Modeling and experimental data,” IEEE Trans Biomed Eng. 43, 857–865. 10.1109/10.508549 9216159

[29] Kasturi, K. , Loizou, P. C. , Dorman, M. , and Spahr, T. (2002). “ The intelligibility of speech with ‘holes' in the spectrum,” J. Acoust. Soc. Am. 112, 1102–1111. 10.1121/1.1498855 12243158

[30] Koch, D. B. , Osberger, M. J. , Segel, P. , and Kessler, D. (2004). “ HiResolution and conventional sound processing in the HiResolution bionic ear: Using appropriate outcome measures to assess speech recognition ability,” Audiol. Neurootol. 9, 214–223. 10.1159/000078391 15205549

[31] Levitt, H. (1971). “ Transformed up-down methods in psychoacoustics,” J. Acoust. Soc. Am. 49(Suppl 2), 467–477. 10.1121/1.1912375 5541744

[32] Litvak, L. M. , Spahr, A. J. , Saoji, A. A. , and Fridman, G. Y. (2007). “ Relationship between perception of spectral ripple and speech recognition in cochlear implant and vocoder listeners,” J. Acoust. Soc. Am. 122, 982–991. 10.1121/1.2749413 17672646

[33] Loizou, P. C. (2006). “ Speech processing in vocoder-centric cochlear implants,” Adv. Otorhinolaryngol. 64, 109–143. 16891839 10.1159/000094648

[34] Long, C. J. , Holden, T. A. , McClelland, G. H. , Parkinson, W. S. , Shelton, C. , Kelsall, D. C. , and Smith, Z. M. (2014). “ Examining the electro-neural interface of cochlear implant users using psychophysics, CT scans, and speech understanding,” J. Assoc. Res. Otolaryngol. 15, 293–304. 10.1007/s10162-013-0437-5 24477546 PMC3946134

[35] Miller, G. A. , and Nicely, P. E. (1955). “ An analysis of perceptual confusions among some English consonants,” J. Acoust. Soc. Am. 27, 338–352. 10.1121/1.1907526

[36] Miura, M. , Sando, I. , Hirsch, B. E. , and Orita, Y. (2002). “ Analysis of spiral ganglion cell populations in children with normal and pathological ears,” Ann. Otol. Rhinol. Laryngol. 111, 1059–1065. 10.1177/000348940211101201 12498365

[37] Nearey, T. M. , and Assmann, P. F. (1986). “ Modeling the role of inherent spectral change in vowel identification,” J. Acoust. Soc. Am. 80, 1297–1308. 10.1121/1.394433

[38] Noble, J. H. , Labadie, R. F. , Gifford, R. H. , and Dawant, B. M. (2013). “ Image-guidance enables new methods for customizing cochlear implant stimulation strategies,” IEEE Trans. Neural Syst. Rehabil. Eng. 21, 820–829. 10.1109/TNSRE.2013.2253333 23529109 PMC3769452

[39] Peterson, G. E. , and Barney, H. L. (1952). “ Control methods used in a study of the vowels,” J. Acoust. Soc. Am. 24, 175–184. 10.1121/1.1906875

[40] Remus, J. J. , Throckmorton, C. S. , and Collins, L. M. (2007). “ Expediting the identification of impaired channels in cochlear implants via analysis of speech-based confusion matrices,” IEEE Trans Biomed Eng. 54, 2193–2204. 10.1109/TBME.2007.908336 18075035

[41] Sagi, E. , Meyer, T. A. , Kaiser, A. R. , Teoh, S. W. , and Svirsky, M. A. (2010). “ A mathematical model of vowel identification by users of cochlear implants,” J. Acoust. Soc. Am. 127, 1069–1083. 10.1121/1.3277215 20136228 PMC2830268

[42] Shannon, R. V. , Galvin, J. J. , and Başkent, D. (2002). “ Holes in hearing,” J. Assoc. Res. Otolaryngol. 3, 185–199. 10.1007/s101620020021 12162368 PMC3202404

[43] Shannon, R. V. , Zeng, F. G. , Kamath, V. , Wygonski, J. , and Ekelid, M. (1995). “ Speech recognition with primarily temporal cues,” Science 270, 303–304. 10.1126/science.270.5234.303 7569981

[44] Shepard, R. N. (1972). “ Psychological representation of speech sounds,” in *Human Communication: A Unified View*, edited by David E. and Denes D. P. ( Cambridge University Press, Cambridge, UK), pp. 67–113.

[45] Stickney, G. S. , Loizou, P. C. , Mishra, L. N. , Assmann, P. F. , Shannon, R. V. , and Opie, J. M. (2006). “ Effects of electrode design and configuration on channel interactions,” Hear. Res. 211, 33–45. 10.1016/j.heares.2005.08.008 16338109

[46] Syrdal, A. K. , and Gopal, H. S. (1986). “ A perceptual model of vowel recognition based on the auditory representation of American English vowels,” J. Acoust. Soc. Am. 79, 1086–1100. 10.1121/1.393381 3700864

[47] ter Keurs, M. , Festen, J. M. , and Plomp, R. (1992). “ Effect of spectral envelope smearing on speech reception. I,” J. Acoust. Soc. Am. 91, 2872–2880. 10.1121/1.402950 1629480

[48] Throckmorton, C. S. , and Collins, L. M. (2002). “ The effect of channel interactions on speech recognition in cochlear implant subjects: Predictions from an acoustic model,” J. Acoust. Soc. Am. 112, 285–296. 10.1121/1.1482073 12141354

[49] Wang, M. D. , and Bilger, R. C. (1973). “ Consonant confusions in noise: A study of perceptual features,” J. Acoust. Soc. Am. 54, 1248–1266. 10.1121/1.1914417 4765809

[50] White, M. W. , Merzenich, M. M. , and Gardi, J. N. (1984). “ Multichannel cochlear implants. Channel interactions and processor design,” Arch Otolaryngol. 110, 493–501. 10.1001/archotol.1984.00800340005002 6547597

[51] Winn, M. B. , Chatterjee, M. , and Idsardi, W. J. (2012). “ The use of acoustic cues for phonetic identification: Effects of spectral degradation and electric hearing,” J. Acoust. Soc. Am. 131, 1465–1479. 10.1121/1.3672705 22352517 PMC3292615

[52] Winn, M. B. , Edwards, J. R. , and Litovsky, R. Y. (2015). “ The impact of auditory spectral resolution on listening effort revealed by pupil dilation,” Ear Hear 36, e153–165. 10.1097/AUD.0000000000000145 25654299 PMC4478109

[53] Won, J. H. , Jones, G. L. , Moon, I. J. , and Rubinstein, J. T. (2015). “ Spectral and temporal analysis of simulated dead regions in cochlear implants,” J. Assoc. Res. Otolaryngol. 16, 285–307. 10.1007/s10162-014-0502-8 25740402 PMC4368650

[54] Wright, R. , and Souza, P. (2012). “ Comparing identification of standardized and regionally valid vowels,” J. Speech Lang. Hear. Res. 55, 182–193. 10.1044/1092-4388(2011/10-0278) 22199181 PMC3288672

[55] Xu, L. , Thompson, C. S. , and Pfingst, B. E. (2005). “ Relative contributions of spectral and temporal cues for phoneme recognition,” J. Acoust. Soc. Am. 117, 3255–3267. 10.1121/1.1886405 15957791 PMC1414641

[56] Zaar, J. , and Dau, T. (2015). “ Sources of variability in consonant perception of normal-hearing listeners,” J. Acoust. Soc. Am. 138, 1253–1267. 10.1121/1.4928142 26428764

[57] Zwolan, T. A. , Collins, L. M. , and Wakefield, G. H. (1997). “ Electrode discrimination and speech recognition in postlingually deafened adult cochlear implant subjects,” J. Acoust. Soc. Am. 102, 3673–3685. 10.1121/1.420401 9407659

